# A new method for identifying and evaluating depressive disorders in young people based on cognitive neurocomputing: an exploratory study

**DOI:** 10.3389/fncom.2025.1555416

**Published:** 2025-02-25

**Authors:** Jiakang Liu, Kai Li, Shuwu Li, Shangjun Liu, Chen Wang, Shouqiang Huang, Yuting Tu, Bo Wang, Pengpeng Zhang, Yuntian Luo, Guanqun Sun, Tong Chen

**Affiliations:** ^1^School of Medical Technology and Information Engineering, Zhejiang Chinese Medical University, Hangzhou, China; ^2^School of Information Engineering, Hangzhou Medical College, Hangzhou, China; ^3^Zhejiang Engineering Research Center for Brain Cognition and Brain Diseases Digital Medical Instruments, Hangzhou Medical College, Hangzhou, China; ^4^Department of Medical Psychology, First Medical Center of Chinese PLA General Hospital, Beijing, China; ^5^Department of Neurology, Second Medical Center of Chinese PLA General Hospital, Beijing, China

**Keywords:** depressive disorders, youth, cognitive function, cognitive impairment, digital biomarkers, identification, evaluation

## Abstract

**Background:**

Depressive disorders are one of the most common mental disorders among young people. However, there is still a lack of objective means to identify and evaluate young people with depressive disorders quickly. Cognitive impairment is one of the core characteristics of depressive disorders, which is of great value in the identification and evaluation of young people with depressive disorders.

**Methods:**

This study proposes a new method for identifying and evaluating depressive disorders in young people based on cognitive neurocomputing. The method evaluates cognitive impairments such as reduced attention, executive dysfunction, and slowed information processing speed that may exist in the youth depressive disorder population through an independently designed digital evaluation paradigm. It also mines digital biomarkers that can effectively identify these cognitive impairments. A total of 50 young patients with depressive disorders and 47 healthy controls were included in this study to validate the method’s identification and evaluation capability.

**Results:**

The differences analysis results showed that the digital biomarkers of cognitive function on attention, executive function, and information processing speed extracted in this study were significantly different between young depressive disorder patients and healthy controls. Through stepwise regression analysis, four digital biomarkers of cognitive function were finally screened. The area under the curve for them to jointly distinguish patients with depressive disorders from healthy controls was 0.927.

**Conclusion:**

This new method rapidly characterizes and quantifies cognitive impairment in young people with depressive disorders. It provides a new way for organizations, such as schools, to quickly identify and evaluate the population of young people with depressive disorders based on human-computer interaction.

## 1 Introduction

Depressive disorders are one of the most common mental disorders, and youth are at high risk for depressive disorders (Gore et al., 2011). According to statistics, about 34% of adolescents worldwide suffer from depressive disorders (Shorey et al., 2022). In recent years, the prevalence of depressive disorders in the youth population has been increasing, and in severe cases, suicidal behaviors may occur, which brings a huge burden to individuals, families, and society (Bukstein, 2022; Daly, 2022). Therefore, accurately identifying and evaluating the population of youth with depressive disorders is of great significance in reducing the prevalence of depressive disorders in this group and comprehensively improving their mental health. Currently, the identification and evaluation of the depressive disorder population relies primarily on depression scales such as the Self-Rating Depression Scale and the Patient Health Questionnaire-9 (PHQ-9) (Zung, 1965; Kroenke et al., 2001). However, these questionnaire measures have shortcomings such as strong subjectivity of the test content, lack of objective quantitative indicators, fixed content, and strong antagonism of repeated measurements, which make it difficult to meet the needs of organizations such as schools for the identification and evaluation of depressive disorders on a large scale. Therefore, there is an urgent need to find a new objective, rapid, accurate method for the large-scale identification and evaluation of young people with depressive disorders.

Cognitive impairment is one of the core characteristics of depressive disorders, which is mainly manifested in patients with depressive disorders as impaired attention, executive function, memory, and information processing speed, and persists during the course of depressive disorders (Conradi et al., 2011; Rock et al., 2014; Atique-Ur-Rehman and Neill, 2019). For young patients with depressive disorders, cognitive impairment not only affects the patient’s daily life and work ability, leading to a series of problems such as learning difficulties, reduced work efficiency, and social disorders but also may increase the risk of relapse of depression and lead to the inability of some patients to return to normal social functioning (Porter and Douglas, 2019; Semkovska et al., 2019). In addition, cognitive dysfunction in patients with depressive disorders is closely related to the regression of affective symptoms, while the severity of affective symptoms affects cognitive function to a certain extent, which can lead to a vicious circle of interaction (Airaksinen et al., 2004). Therefore, cognitive impairment is of great value in the identification and evaluation of young people with depressive disorders. Impaired attention in patients with depressive disorders is mainly characterized by difficulty concentrating or maintaining attention and the inability to maintain sustained focus on goals (Keller et al., 2019). Impaired executive function is mainly characterized by decreased cognitive flexibility, impaired response inhibition, and decreased decision-making ability (Alves et al., 2014). Slowed information processing is primarily characterized by sluggishness of the brain, slower responses, and the need to spend more time processing information (Nuño et al., 2021). In addition, studies have found that cognitive impairments such as executive dysfunction can be used as relevant predictors of depressive disorders (Airaksinen et al., 2007; Letkiewicz et al., 2014). Therefore, based on the above cognitive impairment characteristics of patients with depressive disorders, accurate quantitative evaluation of cognitive impairments in attention, information processing speed, and executive function in patients with depressive disorders can provide new ideas for early identification and evaluation of people with depressive disorders.

Currently, the evaluation of cognitive function in patients with depressive disorders relies mainly on evaluation tools such as the Perceived Deficits Questionnaire-Depression and the Digit Symbol Substitution Test (Jaeger and Zaragoza Domingo, 2016; Shi et al., 2017). Although the Perceived Deficits Questionnaire-Depression is easy to administer and less costly, its results are susceptible to the patient’s subjective feelings, emotional state, and current mental state, thus affecting the accuracy of the evaluation. As a standardized neuropsychological test, the Digit Symbol Substitution Test relies primarily on a paper-and-pencil test format, which cannot adequately capture subtle changes in dynamic cognitive processes. Therefore, there is a need to find a more objective and accurate evaluation method. Digital biomarkers are defined as physiological and behavioral data collected and measured by digital devices, which have many advantages such as objectivity, quantifiability, and fine-grained depiction of complex processes (Kourtis et al., 2019; Vasudevan et al., 2022). It can effectively compensate for the shortcomings of traditional evaluation methods that are highly subjective and cannot accurately quantify the dynamic process of cognition. In addition, studies have found that digital biomarkers are highly sensitive to detecting cognitive changes (Dagum, 2018; Meier et al., 2021). Therefore, digital biomarkers are expected to become an important tool for the evaluation of cognitive function in patients with depressive disorders.

Studies have shown that cognitive task-based digital biomarker evaluation methods have good potential for identifying and evaluating depressive disorders. Mandryk et al. designed a digital evaluation tool based on three cognitive tasks, and the results showed that the use of digital biomarkers from cognitive tasks allowed remote evaluation of depression (Mandryk et al., 2021). Peng et al. used electroencephalography technology to obtain digital biomarkers of patients with depression under cognitive tasks, which indicated that the performance of sustained attention and response inhibition of patients with depression was impaired, and the ability to distinguish patients with depression from healthy controls was 0.94 (Peng et al., 2023). McIntyre et al. developed a cognitive evaluation tool, THINC-it, which contains five digital cognitive tasks. The study found that the digital tool could discriminate between depressed patients from healthy controls and could further quantify cognitive deficits in executive function, working memory, processing speed, and attention in depressed patients (McIntyre et al., 2017). Maalouf et al. used three digitized cognitive tasks from the Cambridge Neuropsychological Test Automated Battery to measure cognitive functioning in patients with depressive disorders. The results showed that executive dysfunction may be a specific marker of depressive disorder (Maalouf et al., 2010). Therefore, a digital cognitive task-based evaluation method can provide an objective and accurate quantitative evaluation of cognitive functioning in patients with depressive disorders and provide strong support for the rapid identification of young people with depressive disorders. In addition, although current cognitive task-based digital biomarker assessment methods have shown good potential in identifying and evaluating depressive disorders, these methods generally suffer from the problem of long test times. Moreover, these methods mostly focus on outcome indicators and lack dynamic analysis of multi-dimensional cognitive components during the task process, making it difficult to quantify cognitive impairment in patients with depressive disorders in a detailed manner. Therefore, this study optimizes the design of cognitive tasks to shorten the test time, quantifies the whole process of cognitive tasks in a fine-grained and continuous manner, and systematically analyzes the temporal change characteristics of key cognitive components such as attention, executive function, and information processing speed, further explores a more rapid and accurate method for identifying and evaluating depressive disorders in young people based on cognitive tasks.

In summary, we propose the following hypothesis: young people with depressive disorder have cognitive impairment, and the impairment can be effectively identified and evaluated based on digital evaluation technology. Based on this hypothesis, we independently designed a new method for identifying and evaluating depressive disorders in young people based on cognitive neurocomputing. The method accurately captures the natural and dynamic cognitive information of young depressive disorder patients during human-computer interaction tasks through a digital evaluation paradigm and characterizes the cognitive impairment of young depressive disorder patients at a fine-grained level. This study aims to reveal the cognitive impairment characteristics of young people with depressive disorders in terms of attention, executive function, and information processing speed, and to provide organizations such as schools with a new way to objectively, quickly, and accurately identify and evaluate young people with depressive disorders.

## 2 Materials and methods

### 2.1 Human-computer interaction evaluation paradigm and digital biomarker design

Based on the research hypothesis that young people with depressive disorders have cognitive impairment and that the impairment can be effectively identified and evaluated by digital evaluation techniques, we designed a digital evaluation paradigm by integrating pupil center cornea reflection techniques. The specific design is as follows.

#### 2.1.1 Prerequisites for paradigm design

**Figure 1:**
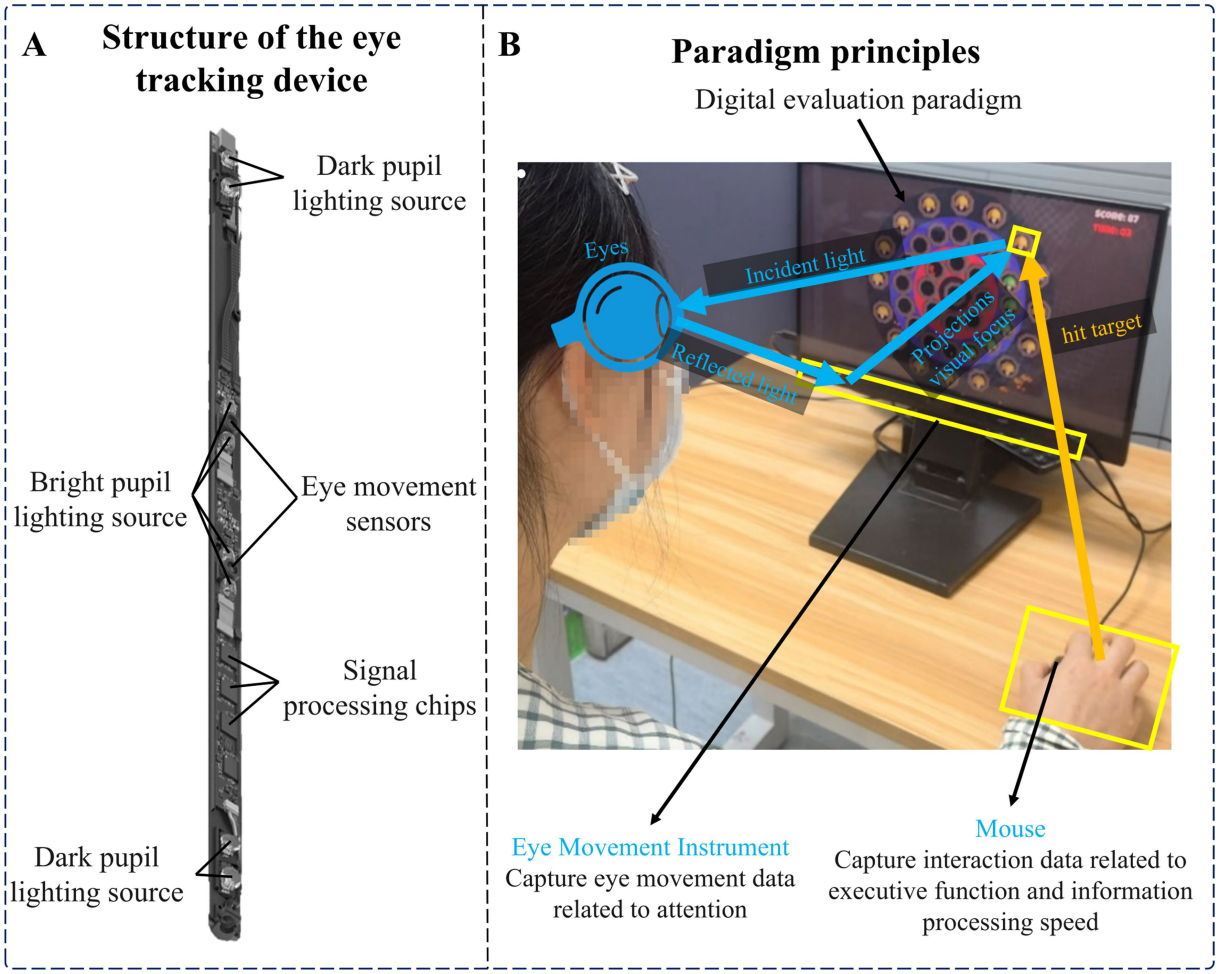
**(A)** Structure of the eye tracker. **(B)** Paradigm principles.

The hardware equipment needed in this experiment included an Intel computer (NUC11PAHi5), a 3,840 × 2,160 pixels interactive display (392 × 250 × 10 mm, 17.3 in.), an eye tracker (Tobii eye tracker 5, sampling rate (SR) was 32 Hz) and a mouse. Among them, the eye tracker consisted of two eye sensors, dark pupil illumination sources, bright pupil illumination sources, and a plurality of signal processing chips, and a plurality of near-infrared light sources were used as reference points for auxiliary analysis. The eye tracker used a modified version of the pupil center corneal reflection eye-tracking technology (US Patent 7572008) (Elvesjo et al., 2009). It can analyze eye position by collecting reflected light to determine the subject’s focus on the screen. Use multi-reference point complementary technology to achieve head trajectory compensation to ensure data accuracy. Refer to Figure 1A for the structural diagram of the eye tracker. The software system involved in this experiment mainly includes a front-end interface, digital evaluation paradigm, back-end system, and database. We built the front-end interface, back-end system, and database through Electron, Vue framework, and MySQL database, and built the digital evaluation paradigm through Unity and eye tracker secondary development interface. See Figure 1B for the schematic diagram of the paradigm principle.

#### 2.1.2 Experimental paradigm design and principle interpretation

Cognitive function is a complex process that can be broken down into multiple areas. The areas most relevant to depressive disorders are attention, executive function, and information processing speed (Alves et al., 2014; Keller et al., 2019; Nuño et al., 2021). Among them, impaired attention is mainly characterized by difficulties in focusing or maintaining attention, and the inability to maintain sustained focus on the target. Impaired executive function is mainly characterized by decreased cognitive flexibility, impaired response inhibition, and decreased decision-making ability. Slowing information processing speed is mainly manifested in slower responses and the need to spend more time processing information. Therefore, we designed a digital evaluation paradigm that included stationary and moving targets to dynamically evaluate these cognitive functions. This paradigm requires subjects to maintain continuous attention to the screen to detect any targets that may appear, and eye movement data collected through eye trackers can assess subjects’ continued attention. In addition, the paradigm requires subjects to quickly identify and respond to targets in a rapidly changing environment. This can not only further test the subjects’ attention allocation and switching abilities, but also assess their cognitive flexibility and response inhibition in executive functions. At the same time, moving targets also increase the difficulty of subjects’ information processing. The time interval between hitting the target collected by the software system can effectively evaluate the information processing speed. A shorter time interval means higher information processing efficiency.

**Figure 2:**
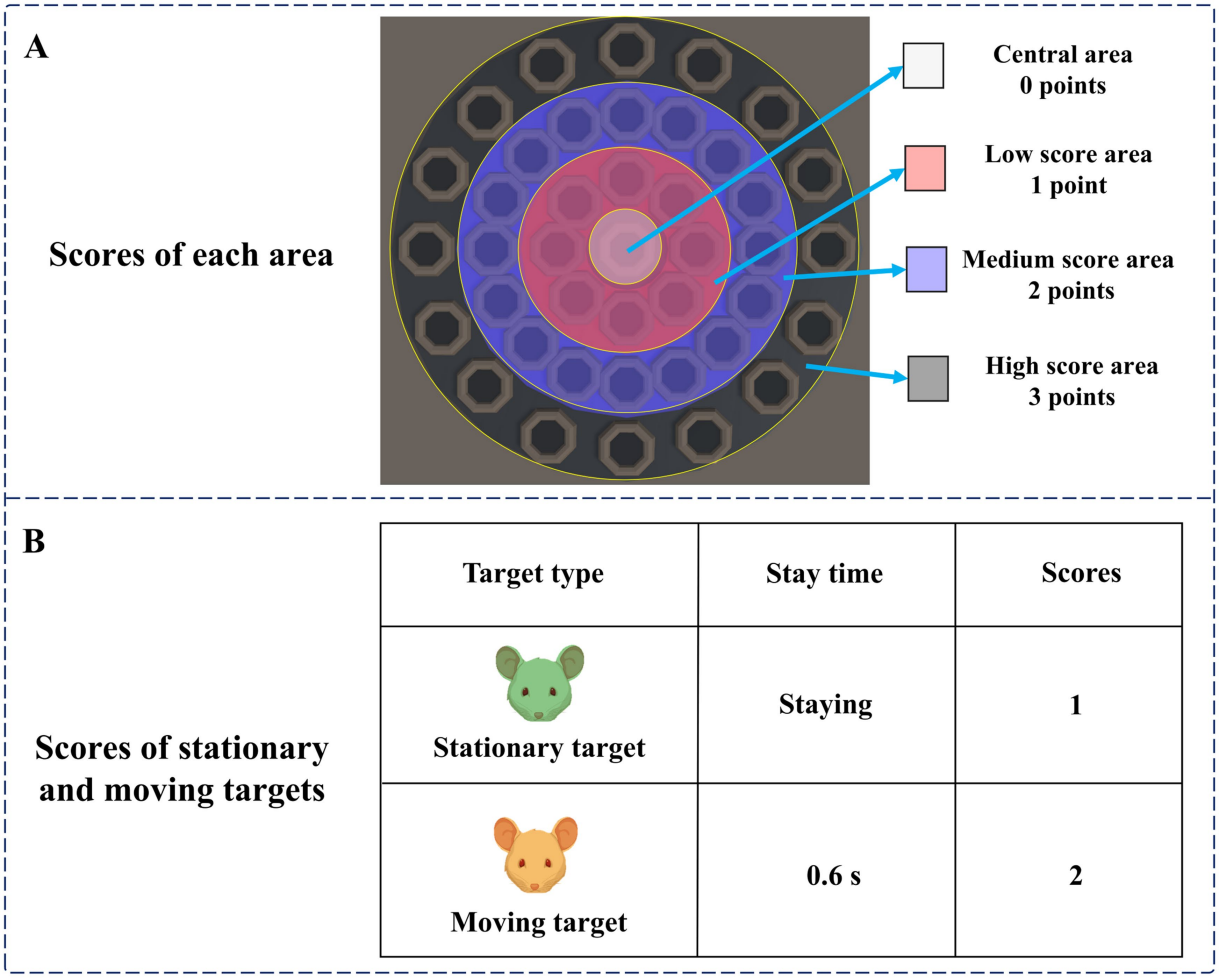
**(A)** Scores of each area. **(B)** Scores of stationary and moving targets.

The specific design of the digital evaluation paradigm is as follows: subjects are required to use the mouse and freely click on a target that appears on the display. After successfully hitting the target, it will disappear and accumulate points. The participant needs to get as many points as possible. The total duration of the paradigm is 15 s, and the cumulative score and countdown will be recorded in real-time in the upper right corner of the display. From inside to outside, the paradigm scene was the central area, the low score area, the medium score area, and the high score area, and the scores of different areas were different. The scores of each area are shown in Figure 2A. There were two types of targets in the paradigm, stationary and moving targets. The scores of stationary and moving targets are shown in Figure 2B.

**Figure 3:**
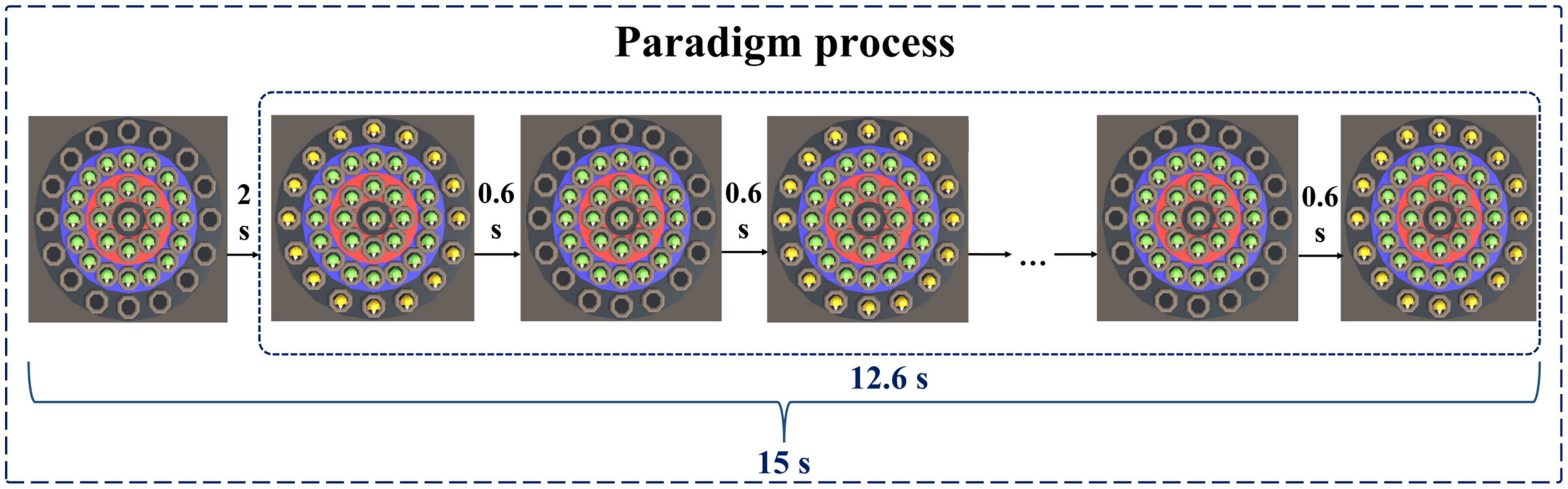
Paradigm process.

At the beginning of the paradigm, stationary targets would appear in the central area, the low score area, and the medium score area. Stationary targets had a base score of 1, with no extra points for appearing in the central area, one extra point for appearing in the low score area, and two extra points for appearing in the medium score area. Therefore, stationary targets that hit the central area, low score area, and medium score area can score 1 point, 2 points, and 3 points, respectively. Two seconds after the paradigm started, moving targets would appear in the high score area. The moving target would blink, that is, it would disappear after 0.6 s, and reappear after an interval of 0.6 s. The blinking would be repeated 10 times. The base score of the moving target was 2 points, and 3 points were added when it appeared in the high score area. Therefore, hitting a moving target in the high score area will earn 5 points. A normal person’s response time to visual stimulation is about 150–300 ms. The brains of people with depressive disorders may have more difficulty processing information, causing them to take longer to respond to stimuli. Therefore, during the paradigm design process, we set parameters such as the residence time and the flashing time interval of the moving target to 0.6 s to ensure that subjects with relatively slow reaction speeds also had enough time to identify and respond, ensuring the effectiveness of the experiment. The paradigm process is shown in Figure 3.

#### 2.1.3 Definition and extraction algorithms for digital biomarkers

Based on the above objective human-computer interaction data, we extracted digital biomarkers of cognitive function via Python (3.10.0). To quantify the cognitive function of subjects in a fine-grained manner, we designed three types of digital biomarkers: “digital biomarkers of attention,” “digital biomarkers of executive function,” and “digital biomarkers of information processing speed.”

Digital biomarkers of attention: Based on an eye tracker, subjects’ attention during the digital evaluation paradigm was measured through pupillary corneal reflex technology. It includes the subjects’ fixation time to different targets in different areas during the task and the number of attention shifts. Task target fixation time is the time of subjects’ attention to different targets (stationary targets, moving targets) in different areas (central area, low score area, medium score area, and high score area) during the task, which is used to characterize subjects’ attention to different targets in different areas. The number of task target attention shifts is the number of times the subject’s gaze shifts from the stationary target to the moving target or from the moving target to the stationary target, which is used to characterize the subject’s attention shifts to the stationary target and the moving target.

Digital biomarkers of executive function: Through human-computer interaction technology, the executive function of subjects during the digital evaluation paradigm is measured. It includes the subject’s task completion score, the total number of executions, the number of completions for different targets in different areas, and the number of uncompletions. The task completion score is the cumulative score of subjects hitting all targets (stationary targets, motor targets). The total number of executions is the number of times the subject used the mouse to click on the screen. The number of uncompletions is the number of times the subject used the mouse to click on the screen but did not hit the target. The above three digital biomarkers were used to characterize the subjects’ overall task performance. The number of task target completions is the number of times the subject used the mouse to hit different targets (stationary targets, moving targets) in different areas (central area, low score area, medium score area, and high score area), which was used to characterize the subject’s execution of different targets in different areas during the task.

Digital biomarker of information processing speed: Through human-computer interaction technology, the time interval for subjects to complete targets during the digital evaluation paradigm task is measured and used to characterize the subjects’ information processing speed.

To facilitate the subsequent digital biomarker mining analysis, we provide a detailed conceptual definition of the three digital biomarkers in the paradigm:

(1) Digital biomarkers of attention

**Figure 4:**
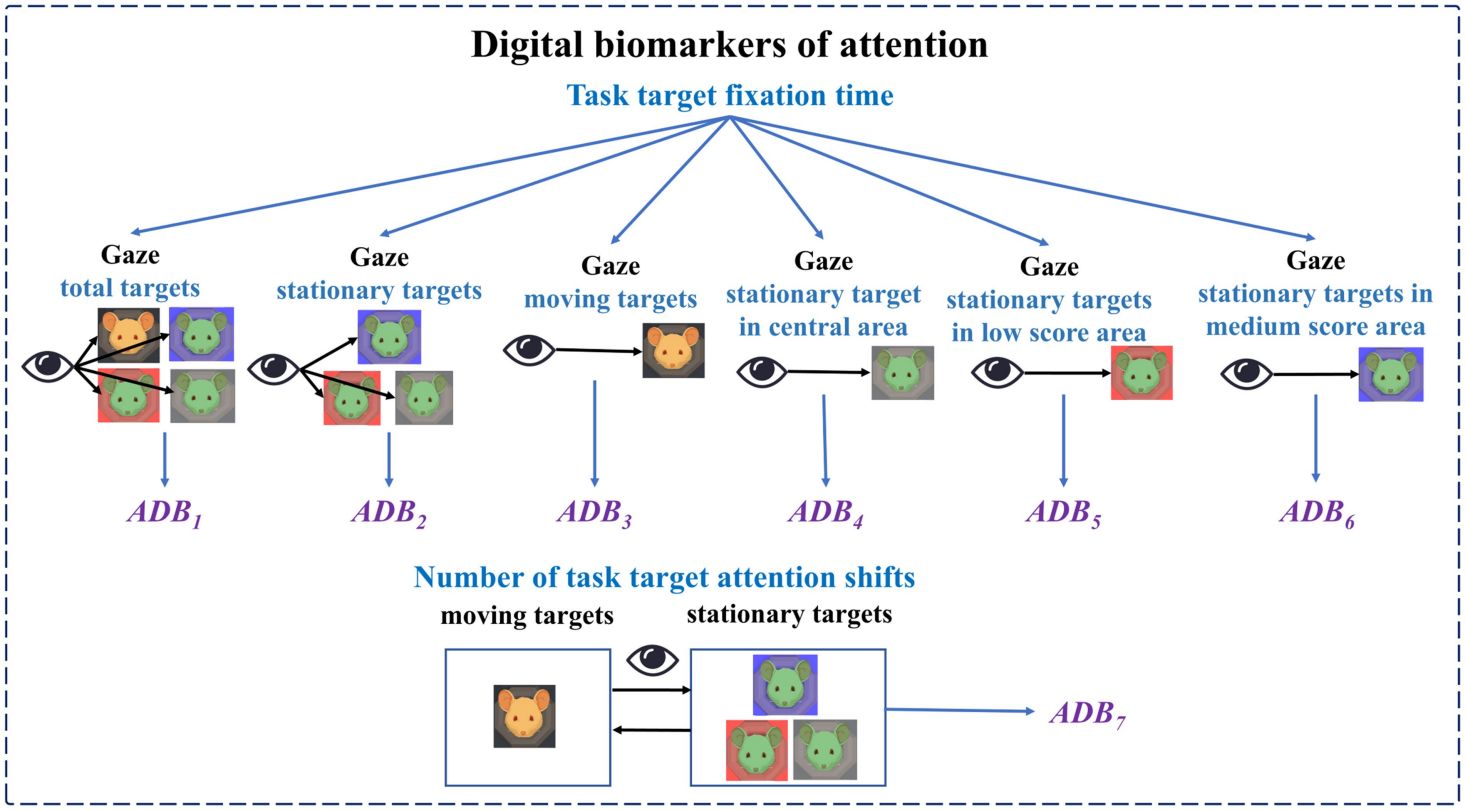
Schematic diagram of digital biomarkers of attention.

The names, abbreviations, and explanations of the digital biomarkers of attention are shown in Table 1. The schematic diagram of the digital biomarkers of attention is shown in Figure 4.

(2) Digital biomarkers of executive function

**Table 1:** Digital biomarkers of attention.

Cognitive impairment	Digital biomarker	Abbreviation	Interpretation
Attention disorders that may be caused by depressive disorders (Keller et al., 2019).	Digital biomarker of attention 1: Task target fixation time	*ADB_1_*	This indicator is used to calculate the cumulative time that subjects gaze at all targets (including stationary and moving targets) during the task (seconds, s).
	Digital biomarker of attention 2: Task target fixation time-static	*ADB_2_*	This indicator is used to calculate the cumulative time that the subjects gaze at stationary targets during the task (seconds, s).
	Digital biomarker of attention 3: Task target fixation time-dynamic	*ADB_3_*	This indicator is used to calculate the cumulative time that subjects gaze at moving targets in the high score area during the task (seconds, s).
	Digital biomarker of attention 4: Task target fixation time-central area	*ADB_4_*	This indicator is used to calculate the cumulative time that subjects gaze at the stationary target in the central area during the task (seconds, s).
	Digital biomarker of attention 5: Task target fixation time-low score area	*ADB_5_*	This metric is used to calculate the cumulative time that subjects gaze at stationary targets in the low score area during the task (seconds, s).
	Digital biomarker of attention 6: Task target fixation time-medium score area	*ADB_6_*	This metric is used to calculate the cumulative time that subjects gaze at stationary targets in the medium score area during the task (seconds, s).
	Digital biomarker of attention 7: Number of task target attention shifts	*ADB_7_*	This indicator is used to calculate the cumulative number of times a subject’s gaze shifts from a stationary target to a moving target or from a moving target to a stationary target during the task (times).

**Figure 5:**
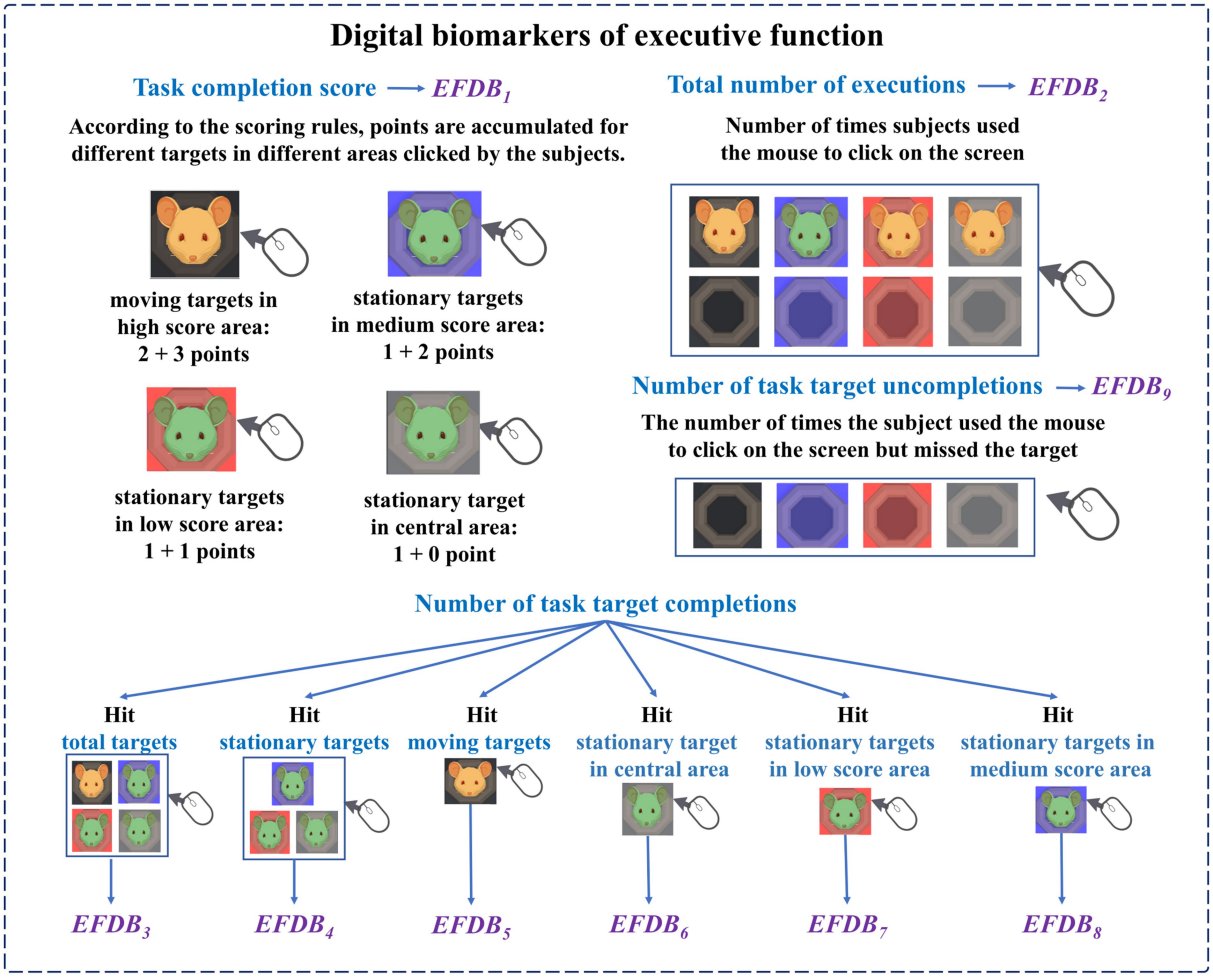
Schematic diagram of digital biomarkers of executive function.

The names, abbreviations, and explanations of the digital biomarkers of executive function are shown in Table 2. The schematic diagram of the digital biomarkers of executive function is shown in Figure 5.

(3) Digital biomarkers of information processing speed

**Table 2:** Digital biomarkers of executive function.

Cognitive impairment	Digital biomarker	Abbreviation	Interpretation
Executive dysfunction that may be caused by depressive disorder (Alves et al., 2014).	Digital biomarkers of executive function 1: Task completion score	*EFDB_1_*	This indicator is used to calculate the cumulative score obtained by subjects hitting all targets (including stationary and moving targets) during the task (score).
	Digital biomarkers of executive function 2: Total number of executions	*EFDB_2_*	This indicator is used to count the number of times the subject uses the mouse to click on the screen during the task, regardless of whether or not they hit the target (times).
	Digital biomarkers of executive function 3: Number of task target completions	*EFDB_3_*	This indicator is used to calculate the cumulative number of times the subject used the mouse to hit all targets (including stationary and moving targets) during the task (times).
	Digital biomarkers of executive function 4: Number of task target completions-static	*EFDB_4_*	This indicator is used to count the number of times the subject used the mouse to hit stationary targets during the task (times).
	Digital biomarkers of executive function 5: Number of task target completions-dynamic	*EFDB_5_*	This indicator is used to calculate the number of times the subject used the mouse to hit moving targets in the high score area during the task (times).
	Digital biomarkers of executive function 6: Number of task target completions-central area	*EFDB_6_*	This indicator is used to calculate the number of times the subject used the mouse to hit the stationary target in the central area during the task (times).
	Digital biomarkers of executive function 7: Number of task target completions-low score area	*EFDB_7_*	This indicator is used to calculate the number of times the subject used the mouse to hit stationary targets in the low score area during the task (times).
	Digital biomarkers of executive function 8: Number of task target completions-medium score area	*EFDB_8_*	This indicator is used to calculate the number of times the subject used the mouse to hit stationary targets in the medium score area during the task (times).
	Digital biomarkers of executive function 9: Number of task target uncompletions	*EFDB_9_*	This indicator is used to calculate the number of times the subject used the mouse to click on the screen but missed the target during the task (times).

One hit on the target is regarded as one target completion.

**Figure 6:**
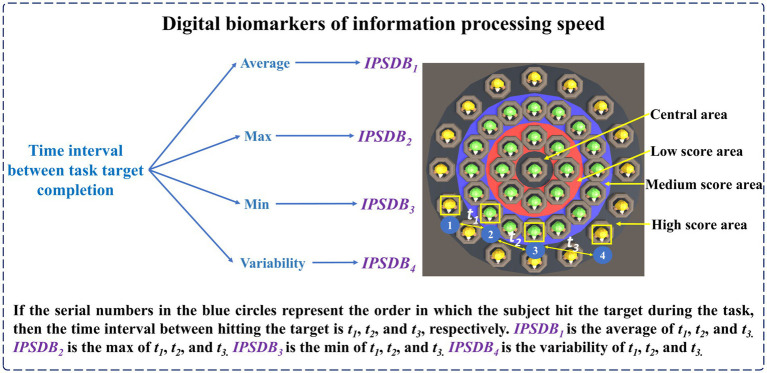
Schematic diagram of digital biomarkers of information processing speed.

The names, abbreviations, and explanations of the digital biomarkers of information processing speed are shown in Table 3. The schematic diagram of the digital biomarkers of information processing speed is shown in Figure 6.

**Table 3:** Digital biomarkers of information processing speed.

Cognitive impairment	Digital biomarker	Abbreviation	Interpretation
Deficiencies in information processing speed that may be caused by depressive disorders (Nuño et al., 2021).	Digital biomarker of information processing speed 1: Average time interval between task target completion	*IPSDB_1_*	This indicator is used to calculate the average of the time interval between the subject using the mouse to hit one target and the next target during the task (seconds, s).
	Digital biomarker of information processing speed 2: Maximum time interval between task target completion	*IPSDB_2_*	This indicator is used to calculate the maximum time interval between the subject using the mouse to hit one target and the next target during the task (seconds, s).
	Digital biomarker of information processing speed 3: Minimum time interval between task target completion	*IPSDB_3_*	This indicator is used to calculate the minimum time interval between the subject using the mouse to hit one target and the next target during the task (seconds, s).
	Digital biomarker of information processing speed 4: Variability of time interval between task target completion	*IPSDB_4_*	This indicator is used to calculate the coefficient of variation of the time interval between when the subject uses the mouse to hit one target and when he hits the next target during the task.

One hit on the target is regarded as one target completion.

We designed intelligent analysis algorithms for the above digital biomarkers of cognitive function as follows:

We assumed that a total of *S* eye movement coordinates of the subject were captured during the digital evaluation paradigm. The *s-*th eye movement coordinate was (*EX_s_*, *EY_s_*), 1≤s≤S, s∈N. The subject clicked on the screen *Q* times, i.e., the total number of executions (*EFDB_2_*) = *Q*. The subject hit the target *R* times, i.e., the number of task target completions (*EFDB_3_*) = *R*. The time interval between the *r*-th task target completion and the (*r +* 1)-th task target completion was *t_r_* (1≤r≤R−1<Q, r∈N).

According to the subject’s eye movement staying information on each type of target object, we calculated the relevant indicators of task target fixation time and the number of task target attention shifts (*ADB_7_*). The relevant calculation methods are shown in Equations 1-7:


(1) $$\text{Fixation}\left(s,\text{target}\;\text{type},\text{area}\right)=\{\begin{array}{c} 1,\text{True}\\ 0,\text{False} \end{array}$$

(2) $$\text{AD}B_{6}=\sum_{s=1}^{S}\frac{\text{Fixation}\left(s,ST,MSA\right)}{SR}\left(1\leq s\leq S,s\in N\right)$$

(3) $$\text{AD}B_{2}=\text{AD}B_{4}+\text{AD}B_{5}+\text{AD}B_{6}$$

(4) $$\text{AD}B_{3}=\sum_{s=1}^{S}\frac{\text{Fixation}\left(s,MT,HSA\right)}{SR}\left(1\leq s\leq S,s\in N\right)$$

(5) $$\text{AD}B_{1}=\text{AD}B_{2}+\text{AD}B_{3}$$

(6) $$\text{Shift}\left(s,s+1\right)=\{\begin{array}{c} 0,\text{True}\\ 1,\text{False} \end{array}$$

(7) $$\text{AD}B_{7}=\sum_{s=1}^{S-1}\text{Shift}\left(s,s+1\right)\left(1\leq s\leq S-1,s\in N\right)$$

Equation 1 was used to determine whether the *s*-th eye movement coordinate stays on a certain target object in a certain area. The *target type* means different types of targets, including stationary targets (*ST*) and moving targets (*MT*). The *area* means different types of area, including central area (*CA*), low score area (*LSA*), medium score area (*MSA*), and high score area (*HSA*). The *SR* in Equation 2 means eye movement sampling frequency. This equation was used to calculate the fixation time of the subject’s gaze on the stationary target in the medium score area, which was denoted as the task target fixation time-medium score area (*ADB_6_*). The calculation methods for the task target fixation time-central area (*ADB_4_*) and task target fixation time-low score area (*ADB_5_*) were similar to those of *ADB_6_*. Equation 6 was used to determine whether the target objects that the subject was paying attention to were of the same type when the *s*-th eye movement coordinate and the (*s* + 1)-th eye movement coordinate were collected. If the target objects were not of the same type, the result of Equation 6 was equal to 1.

For the calculation methods of the number of task target completions (*EFDB_3_*), the number of task target completions-static (*EFDB_4_*), and the number of task target uncompletions (*EFDB_9_*) are shown in Equations 8-10:


(8) $$EFDB_{3}=EFDB_{4}+EFDB_{5}$$

(9) $$EFDB_{4}=EFDB_{6}+EFDB_{7}+EFDB_{8}$$

(10) $$EFDB_{9}=EFDB_{2}-EFDB_{3}$$

The calculation equations for the average time interval between task target completion (*IPSDB_1_*), the maximum time interval between task target completion (*IPSDB_2_*), the minimum time interval between task target completion (*IPSDB_3_*), and the variability of time interval between task target completion (*IPSDB_4_*) are shown in Equations 11-15:


(11) $$\text{IPSD}B_{1}=\frac{\sum_{r=1}^{R-1}t_{r}}{R-1}\left(1\leq r\leq R-1,r\in N\right)$$

(12) $$\text{IPSD}B_{2}=\max_{1\leq r\leq R-1}t_{r}$$

(13) $$\text{IPSD}B_{3}=\min_{1\leq r\leq R-1}t_{r}$$

(14) $$\sigma_{t}=\sqrt{\frac{\sum_{r=1}^{R-1}{\left(t_{r}-\text{IPSD}B_{1}\right)}^{2}}{R-1}}$$

(15) $$\text{IPSD}B_{4}=\frac{\sigma_{t}}{\text{IPSD}B_{1}}$$

The $\sigma_{t}$ in Equation 15 was the standard deviation of the time interval between task target completion.

#### 2.1.4 Standardization of experimental paradigms

Subjects conducted paradigm evaluations in a quiet room to prevent noisy environments from affecting experimental results. Subjects sat in a comfortable chair, 80 cm away from the monitor. Ensure that subjects can clearly see the screen and operate the mouse conveniently to complete the paradigm interaction, thus avoiding the influence of visual interference or inconvenient operation on the experimental results. Before the paradigm evaluation, subjects will use the eye tracker calibration software to calibrate their eyes to ensure the accuracy of eye movement data.

### 2.2 Experimental setup

To validate the efficacy of the new method for identifying and evaluating depressive disorders in young people based on cognitive neurocomputing, we conducted a cross-sectional study in Zhejiang Province.

#### 2.2.1 Subjects

This study recruited 110 subjects from universities in Hangzhou City, Zhejiang Province. Ten of them were not included in the experiment because they did not meet the criteria. Finally, 100 people were included in the experiment, including 51 patients with depressive disorders and 49 healthy controls matched for age and sex. Subjects were divided into depressive disorder group (DD group, *n* = 51, where “*n*” represents sample size) and healthy control group (HC group, *n* = 49). All subjects underwent clinical evaluation and diagnosis by a psychiatrist. All experimental procedures in this study comply with the Helsinki Declaration and have been approved by the Ethics Committee of Zhejiang Chinese Medical University (Approval Number: 20230627-1). All subjects participated voluntarily. Specific inclusion and exclusion criteria are shown below.

Inclusion criteria for the DD group: (1) meeting the diagnostic criteria for depressive disorders in the 11th edition of the International Classification of Diseases (ICD-11); (2) not taking any psychiatric medication for at least 14 days prior to enrolment; (3) age 15–24, regardless of gender; (4) being able to cooperate in completing the digital evaluation paradigm; (5) signing informed consent.

Inclusion criteria for the HC group: (1) no personal or family history of mental illness; (2) age 15–24, regardless of gender; (3) being able to cooperate in completing the digital evaluation paradigm; (4) signing informed consent.

Exclusion criteria for all subjects: (1) presence of other psychiatric disorders; (2) presence of visual impairment and other serious physical illnesses; (3) history of alcohol or psychoactive substance abuse or dependence; (4) inability to cooperate in completing the paradigm for other reasons.

**Figure 7:**
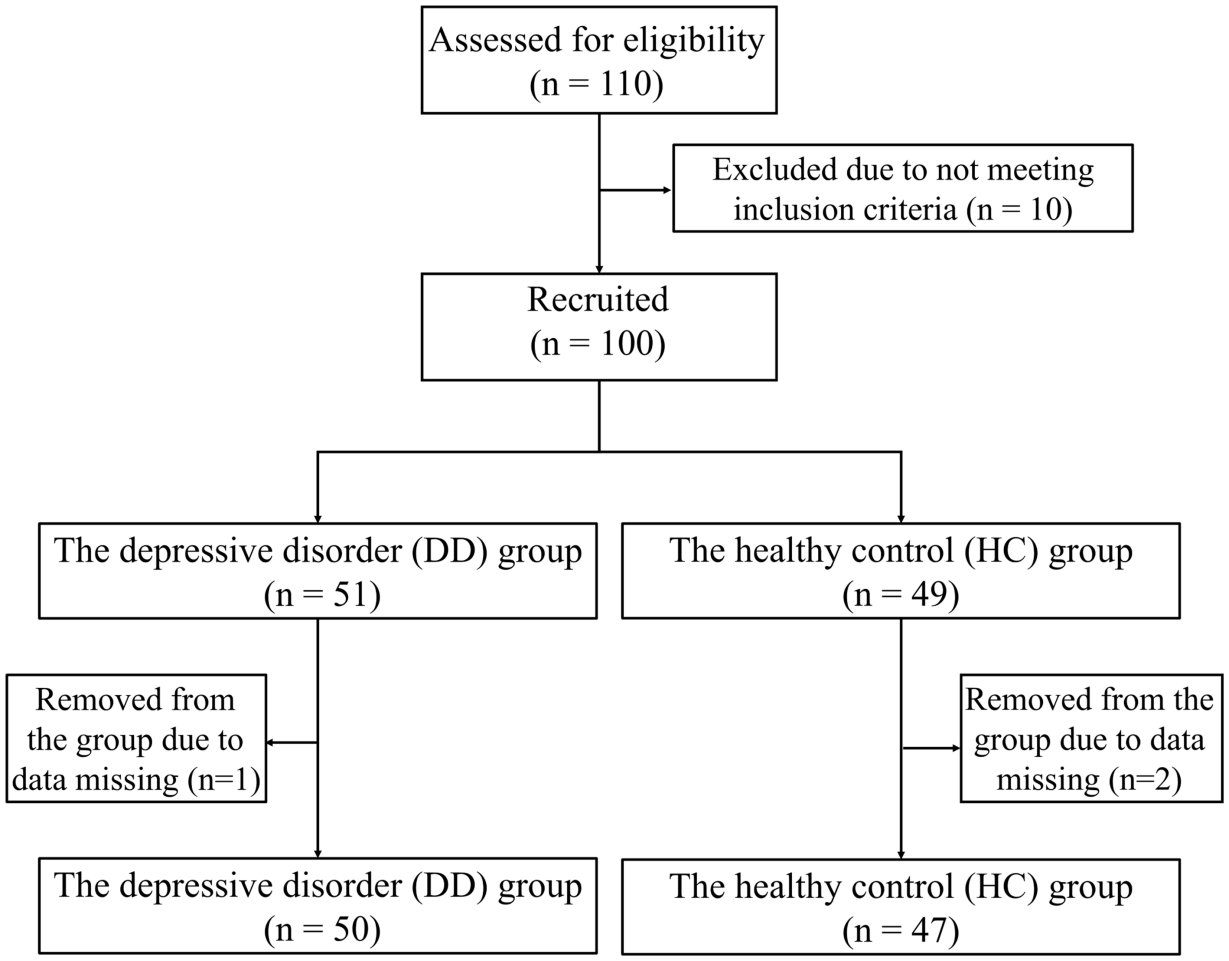
Flow chart of subjects screening.

During the formal experiment, three individuals (one in the DD group and two in the HC group) were excluded due to abnormalities in digital biomarker data collection, resulting in missing data. Ultimately, the effective sample size was 97 individuals, including 50 patients with depressive disorders and 47 healthy controls. The flowchart of the subject screening process is shown in Figure 7. To ensure data consistency, subjects participated in the digital evaluation paradigm on the same day and completed the evaluation of the PHQ-9 scale.

#### 2.2.2 Experimental process

**Figure 8:**
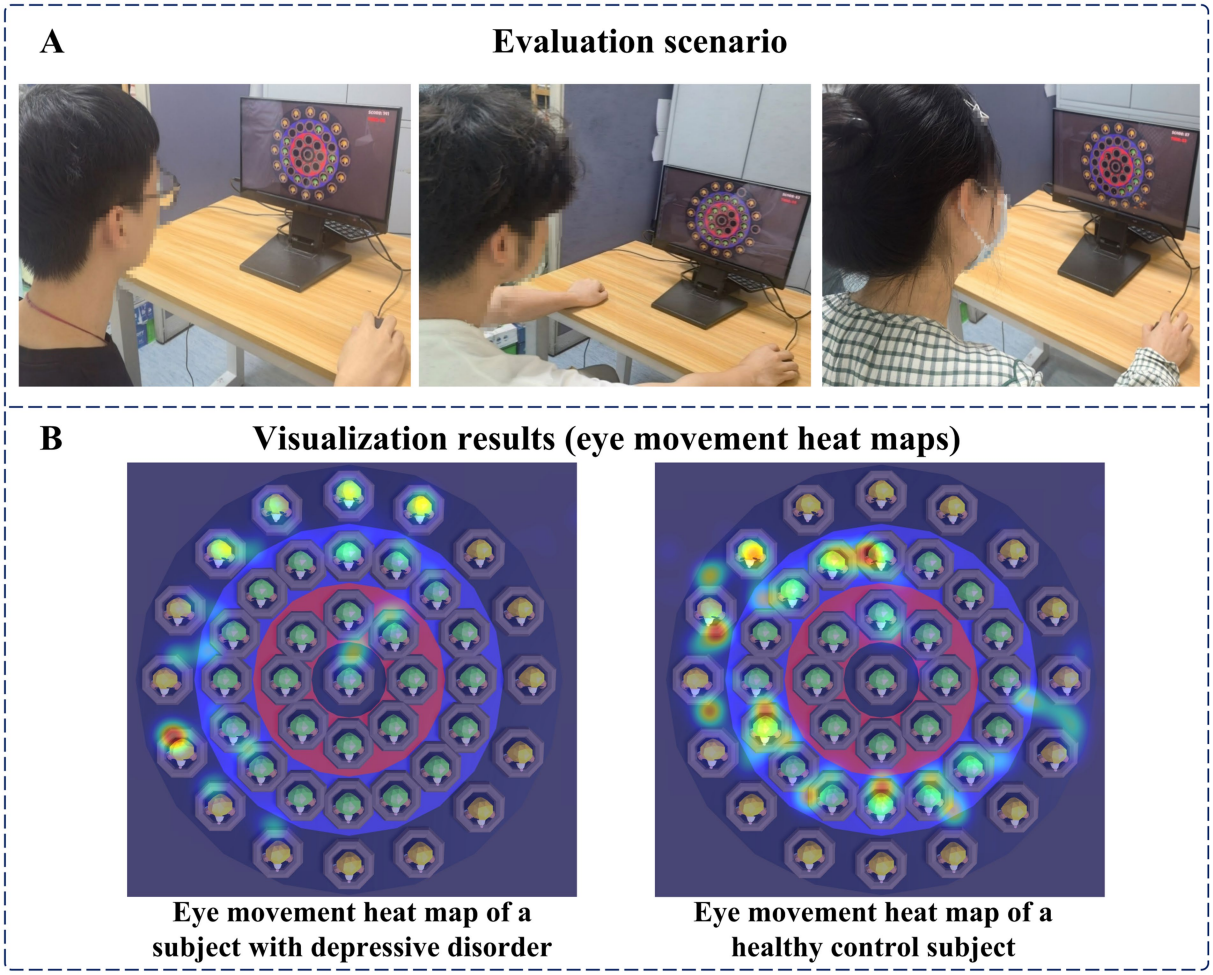
**(A)** Evaluation scenario. **(B)** Visualization results (eye movement heat maps).

Before the digital evaluation paradigm began, subjects were asked to sit in a chair at the table and calibrate their gaze through the calibration software that came with the eye tracker. Afterward, staff would inform subjects about the paradigm process, operating method, and scoring rules, and only after subjects were fully familiar with it would the paradigm experiment formally begin. After the digital evaluation paradigm officially started, subjects needed to use the mouse and click on the targets appearing on the display to get as many points as possible. The evaluation scenario is shown in Figure 8A. In the paradigm evaluation process, we used an eye tracker and a human-computer interaction software system to record the relevant interaction data. At the end of the paradigm, subjects can view the eye movement heat map results to understand their gaze, as shown in Figure 8B.

### 2.3 Data analysis

In this study, SPSS26.0 statistical software was used for data analysis. The chi-square test was used to compare inter-group differences in counting data. The measurement data of normal distribution were presented as mean ± standard deviation, and differences between groups were compared using the independent samples t-test. The measurement data of skewness distribution were presented as median (interquartile range), and differences between groups were compared using the Mann–Whitney U-test. In addition, we used a stepwise binary logistic regression method to screen cognitive digital biomarkers. For the joint evaluation of multiple digital biomarkers of cognitive function, we used a binary logistic regression model to perform a multi-factor analysis. Finally, we plotted receiver operating characteristic (ROC) curves and compared the areas under the curves (AUC) to evaluate the ability of a single cognitive digital biomarker and a combination of multiple cognitive digital biomarkers to distinguish patients with depressive disorders from healthy controls. *p* < 0.05 was considered to indicate a statistically significant difference.

## 3 Results

### 3.1 Demographic and clinical characteristics

The effective sample size of this study was 97 youths, including 50 patients with depressive disorders and 47 healthy controls. They were included in the DD and HC groups, respectively. We analyzed the differences in demographic characteristics and PHQ-9 scale scores between the two groups. The results showed that there was no significant difference between the two groups of subjects in terms of age and gender (*p* > 0.05), while the PHQ-9 scale scores of the DD group were significantly higher than those of the HC group (*p* < 0.05), as shown in Table 4.

**Table 4:** Difference analysis results of demographic and clinical characteristics between DD and HC groups.

	DD Group (*n* = 50)	HC Group (*n* = 47)	*p* value
Age, years	18.00 (1.00)	18.00 (1.00)	0.466
Sex (female/male)	12/38	11/36	0.945
PHQ-9 score**	12.00 (1.50)	1.00 (3.00)	<0.001

**p value < 0.01.

### 3.2 Analysis of digital biomarkers

We then compared all digital biomarkers involved in completing the digital evaluation paradigm between the DD and HC groups. A total of 14 digital biomarkers of cognitive function were found that were significantly different between the DD and HC groups (*p* < 0.05). Among the digital biomarkers of attention, the task target fixation time-static (*ADB_2_*), task target fixation time-low score area (*ADB_5_*), and task target fixation time-medium score area (*ADB_6_*) in the DD group were significantly smaller than those in the HC group. Among the digital biomarkers of executive function, the DD group’s task completion score (*EFDB_1_*), total number of executions (*EFDB_2_*), number of task target completions (*EFDB_3_*), number of task target completions-static (*EFDB_4_*), number of task target completions-dynamic (*EFDB_5_*), number of task target completions-low score area (*EFDB_7_*), number of task target completions-medium score area (*EFDB_8_*), and number of task target uncompletions (*EFDB_9_*) were significantly lower than those of the HC group. Among digital biomarkers of information processing speed, the average time interval between task target completion (*IPSDB_1_*), maximum time interval between task target completion (*IPSDB_2_*), and minimum time interval between task target completion (*IPSDB_3_*) in the DD group were significantly higher than those in the HC group. The results of the complete intergroup differential analysis of digital biomarkers of cognitive function are shown in Table 5.

**Table 5:** Differences analysis results of digital biomarkers of cognitive function.

	DD Group (*n* = 50)	HC Group (*n* = 47)	*p* value
Digital biomarkers of attention			
*ADB_1_*	3.42 ± 1.72	3.61 ± 1.23	0.538
ADB_2_**	0.83 (1.11)	1.25 (1.06)	<0.001
*ADB_3_*	2.50 (2.29)	1.84 (1.34)	0.121
*ADB_4_*	0.00 (0.00)	0.00 (0.19)	0.159
ADB_5_*	0.00 (0.16)	0.22 (0.44)	0.022
ADB_6_**	0.63 (1.02)	1.00 (1.22)	0.002
*ADB_7_*	3.00 (2.25)	2.00 (3.00)	0.053
Digital biomarkers of executive function			
EFDB_1_**	71.02 ± 12.55	85.81 ± 9.79	<0.001
EFDB_2_**	33.50 (16.25)	50.00 (26.00)	<0.001
EFDB_3_**	20.00 (4.00)	24.00 (3.00)	<0.001
EFDB_4_**	12.88 ± 4.34	16.83 ± 4.10	<0.001
EFDB_5_*	7.00 (2.00)	8.00 (4.00)	0.043
*EFDB_6_*	1.00 (1.00)	1.00 (1.00)	0.241
EFDB_7_*	2.00 (2.25)	2.00 (4.00)	0.012
EFDB_8_**	11.00 (5.00)	13.00 (4.00)	<0.001
EFDB_9_**	12.00 (14.25)	24.00 (27.00)	<0.001
Digital biomarkers of information processing speed			
IPSDB_1_**	0.72 (0.13)	0.57 (0.09)	<0.001
IPSDB_2_**	1.58 (0.52)	1.33 (0.37)	0.004
IPSDB_3_**	0.28 (0.09)	0.23 (0.11)	<0.001
*IPSDB_4_*	0.16 (0.12)	0.16 (0.09)	0.166

*p value <0.05; **p value <0.01.

### 3.3 ROC curve analysis for distinguishing patients with depressive disorder from healthy controls

**Figure 9:**
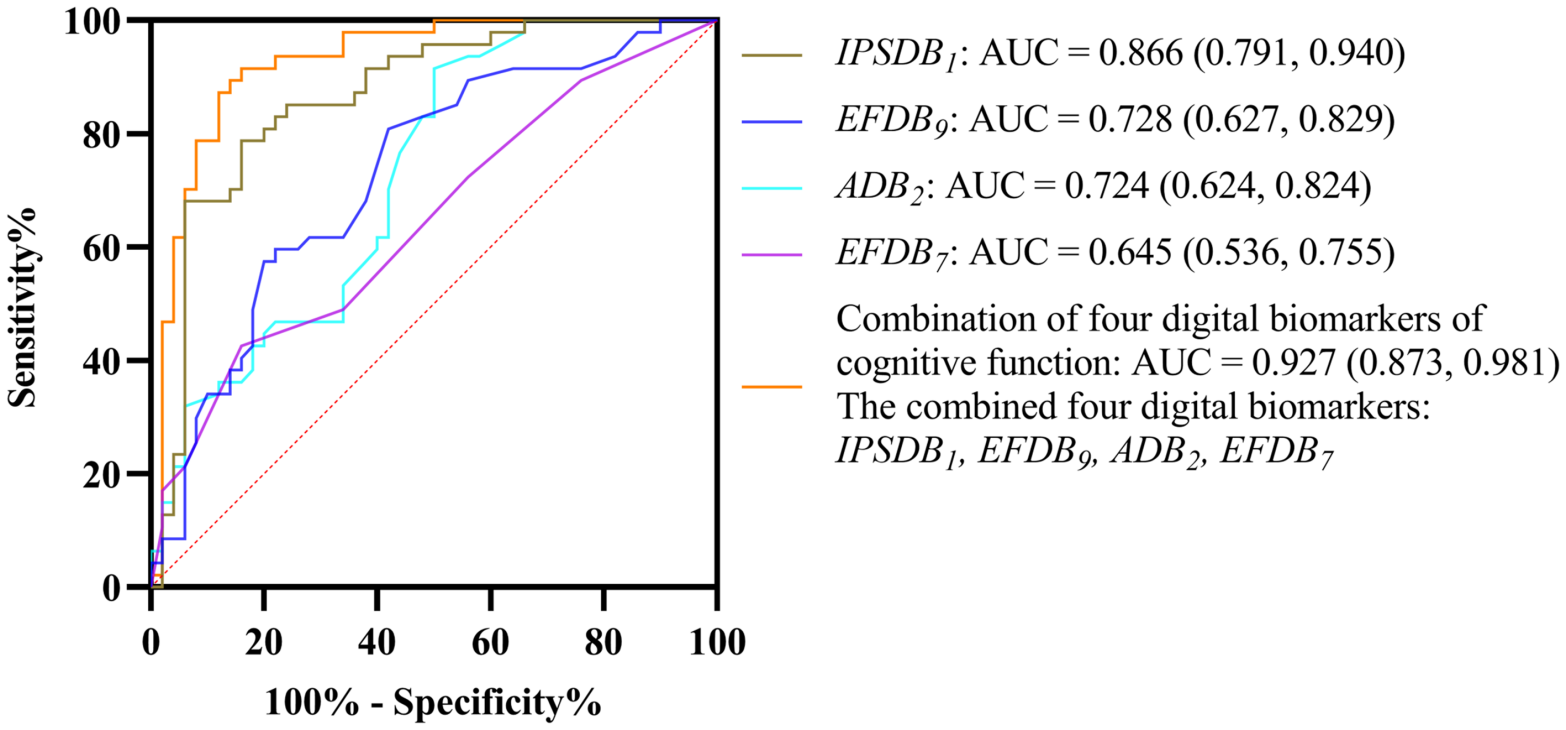
ROC curves, area under the curve, and 95% confidence intervals for four digital biomarkers of cognitive function and their combinations to distinguish DD and HC groups.

Given the limited effective sample size of this study and when too many indicators are included, the model is at risk of overfitting. Therefore, we used a stepwise binary logistic regression method to reduce the dimension of digital biomarkers. In this process, we used whether we had depressive disorder as a dependent variable (binary categorical variable, where 1 represents depressive disorder patients and 0 represents healthy controls), and all digital biomarkers of cognitive function with intergroup differences were included in the initial model as independent variables. Subsequently, we used the forward stepwise regression method to screen variables. In the end, four digital biomarkers of cognitive function were retained: task target fixation time-static (*ADB_2_*), number of task target completions-low score area (*EFDB_7_*), number of task target uncompletions (*EFDB_9_*), and the average time interval between task target completion (*IPSDB_1_*). We then plotted ROC curves and calculated AUC values to evaluate the ability of screened digital biomarkers of cognitive function to distinguish young patients with depressive disorders from healthy controls. The results of ROC curve analysis showed that the AUC of the average time interval between task target completion (*IPSDB_1_*) was 0.866, the AUC of the number of task target uncompletions (*EFDB_9_*) was 0.728, the AUC of task target fixation time-static (*ADB_2_*) was 0.724, and the AUC of number of task target completions-low score area (*EFDB_7_*) was 0.645. The combined AUC value for the above four digital biomarkers was 0.927. The ROC curve, area under the curve, and 95% confidence interval are shown in Figure 9.

## 4 Discussion

In this study, we proposed a new method for identifying and evaluating depressive disorders in young people based on cognitive neurocomputing. This method has the advantages of fast speed, low cost, high intelligence, and strong anti-confrontation. We designed a digital evaluation paradigm to evaluate subjects’ cognitive function and extracted digital biomarkers that can reflect cognitive impairment in young people with depressive disorders. To avoid overfitting, we used a stepwise regression method to reduce the dimensions of the above digital biomarkers. Finally, four digital biomarkers of cognitive function were screened out, namely, task target fixation time-static (*ADB_2_*), number of task target completions-low score area (*EFDB_7_*), number of task target uncompletions (*EFDB_9_*), and the average time interval between task target completion (*IPSDB_1_*). The AUC combined to distinguish young patients with depressive disorders from healthy controls can reach 0.927.

Cognitive impairment in people with depressive disorders is complex, including dysfunction in attention, executive function, and information processing speed. Traditional cognitive function evaluation methods have problems such as long time-consuming and subjectivity and are difficult to quickly and accurately reflect the overall cognitive status of people with depressive disorders. Research has found that cognitive function can be accurately characterized and quantified through specific digital tasks, with high sensitivity and repeatability (Meier et al., 2021). In the digital evaluation paradigm task designed in this study, subjects needed to click quickly and accurately on the target that appeared. This task process requires the mobilization of multiple cognitive functions of the subject’s attention, executive function, and information processing speed. Therefore, the digital evaluation paradigm designed in this study can accurately characterize the cognitive impairment characteristics of young people with depressive disorders and has important scientific significance for the accurate identification and evaluation of this group.

First, the results of this study showed that among the digital biomarkers of attention in the DD group, the task target fixation time-static (*ADB_2_*), task target fixation time-low score area (*ADB_5_*), and task target fixation time-medium score area (*ADB_6_*) were significantly smaller than those in the HC group. However, among the digital biomarkers of information processing speed in the DD group, the average time interval between task target completion (*IPSDB_1_*), maximum time interval between task target completion (*IPSDB_2_*), and minimum time interval between task target completion (*IPSDB_3_*) were significantly higher than those in the HC group. The above findings suggest that young people with depressive disorders may have decreased attention span and slower information processing compared to the normal population, which is generally consistent with the findings of previous studies (Atique-Ur-Rehman and Neill, 2019). Current cognitive psychology research finds that people with depression are often accompanied by negative cognitive bias, and individuals may fall into thinking patterns such as self-reflection and emotional rumination, which makes it difficult to focus on key external information when performing tasks, requiring more time to screen and identify effective information, thus affecting the overall information processing speed (Wagner et al., 2015; Schwert et al., 2017; Owens et al., 2021). Therefore, the DD group may have decreased attention and slowed down information processing during paradigm evaluation due to excessive self-focus and rumination, which ultimately manifested in shorter target fixation times and longer target completion intervals.

Secondly, we found that among the digital biomarkers of executive function in the DD group, the task completion score (*EFDB_1_*), the total number of executions (*EFDB_2_*), the number of task target completions (*EFDB_3_*), the number of task target completions-static (*EFDB_4_*), the number of task target completions-dynamic (*EFDB_5_*), the number of task target completions-low score area (*EFDB_7_*), and the number of task target completions-medium score area (*EFDB_8_*) were significantly lower than those in the HC group. These findings are generally consistent with McIntyre et al. (2017). It suggests that young people with depressive disorders may have executive dysfunction. Executive function is the general term for a series of complex cognitive processes involved in an individual’s achievement of goal-oriented behavior, including inhibition and control, cognitive flexibility, and other aspects. These aspects interact in concert to ensure that individuals can effectively plan, organize, monitor, and adjust their behaviors to achieve goals (Diamond, 2013). Currently, many studies have shown that executive dysfunction in people with depressive disorders is mainly related to the frontal cortex and anterior cingulate region, especially the dorsolateral prefrontal cortex (Salehinejad et al., 2021; Tian et al., 2025). The dorsolateral prefrontal cortex (DLPFC) plays a key role in multiple executive functions such as response inhibition, cognitive flexibility, working memory, planning, and abstract reasoning (Jung et al., 2022). A functional magnetic resonance imaging study showed that patients with depression had increased functional connectivity in the DLPFC and poorer inhibitory control compared to healthy controls (Zhang et al., 2022). In addition, several studies have shown that transcranial magnetic stimulation of the DLPFC in depression patients can effectively improve their executive functions such as response inhibition and cognitive flexibility (Asgharian Asl and Vaghef, 2022; Hernández-Sauret et al., 2024). Therefore, young people with depressive disorders may have executive dysfunction due to DLPFC dysfunction, which ultimately results in abnormalities in digital biomarkers of executive function during paradigm evaluation.

It is worth noting that the number of task target uncompletions (*EFDB_9_*) in the DD group was significantly lower than that in the HC group, which is inconsistent with previous studies that found that patients with depression had a greater number of errors or unfinished times than normal people (Hoffmann et al., 2017). Analyzing the reason, it may be that the paradigm we designed requires subjects to click on the target as many times as possible to get a higher score. In pursuit of higher scores, people in the HC group tend to click quickly and frequently, which increases the risk of missing the target to some extent and leads to an increase in the number of missed goals. However, people in the DD group may have fewer attempts to explore and take advantage of opportunities during tasks due to lack of anhedonia and reduced motivation to participate in activities (Treadway and Zald, 2011; Treadway et al., 2012; Cooper et al., 2018). As a result, the number of task target uncompletions in the DD group was significantly smaller than that in the HC group.

In addition, this study innovatively proposes a new method for identifying and evaluating depressive disorders in young people based on cognitive neurocomputing, which has significant advantages compared to traditional methods for identifying and evaluating depression. In terms of testing efficiency, traditional identification and evaluation tools such as the Hamilton Depression Rating Scale require long-term face-to-face communication between professional physicians and patients, which takes 15–20 min. The new method uses a computer to complete the paradigm evaluation, which can be completed in 1 min, greatly shortening the testing time and facilitating large-scale screening or outpatient pre-screening in schools and other organizations. In terms of objectivity and subjectivity, traditional identification and evaluation methods rely on physician judgment and patient subjective factors. Patients’ emotions, ability to express themselves, and individual differences in physicians lead to strong subjectivity in results. The new method is based on objective evaluation data and is not affected by subjective emotions and human biases, and the evaluation is more accurate and objective. In terms of clinical adaptability and cost-effectiveness, traditional identification and evaluation methods rely heavily on professional physicians and are mostly limited to specific locations. The new method requires only ordinary computers and is easy to operate. Non-professionals can help with simple training. It greatly reduces labor costs and can be extended to many areas such as schools and communities. In addition, the existing digital identification methods for depression are mostly achieved by obtaining the behavioral characteristics of the subject’s mobile phones, text content posted on social media, and physiological behavior indicators captured by wearable devices (Eichstaedt et al., 2018; Asare et al., 2022; Mullick et al., 2022). This not only requires long-term monitoring, but also has issues such as data confidentiality and privacy disclosure, and low patient trust and acceptance (Lee et al., 2021). Therefore, the new method proposed in this study not only makes up for the shortcomings of traditional identification and evaluation methods that are time-consuming and highly subjective but also overcomes the problems of long monitoring time, privacy disclosure, and low patient acceptance of existing digital identification methods. It provides a rapid, accurate, intelligent identification and evaluation tool for young people with depressive disorders.

This study also has some limitations. (1) The effective sample size of this study is 97 cases, the total sample size is limited, and only young people aged 18–20 are included. The results of the study may not reflect the situation of the general population with depressive disorders. (2) The paradigm used in the current study is inadequate in evaluating the memory abilities of patients with depressive disorders. (3) Although the digital biomarkers extracted in this study have shown good efficiency in identifying and evaluating depressive disorders, this study did not explore in depth the neurobiological mechanisms in which digital biomarkers may be involved. (4) Although the digital biomarkers extracted in this study showed high AUC values in identifying and evaluating depressive disorders, there may be a risk of overfitting due to the limited study sample size and excessive number of digital biomarkers. Currently, our research conditions are limited. In the future, we will obtain larger-scale data through multi-center cooperation to further verify the generalization capability of the model. Simultaneously, mitigation strategies such as cross-verification and regularization techniques are used to improve the robustness of the model.

## 5 Conclusion

In summary, we put forward the following hypothesis: young people with depressive disorders have cognitive impairment, and digital evaluation techniques can effectively identify and evaluate this impairment. Based on this assumption, we proposed a new method for identifying and evaluating depressive disorders in young people based on cognitive neurocomputing, designed a digital evaluation paradigm for evaluating cognitive function, and mined digital biomarkers that can calculate cognitive impairment in young patients with depressive disorders. After preliminary clinical verification, this method can finely characterize and quantify the cognitive impairment of young patients with depressive disorders in terms of attention, executive function, and information processing speed. The ability to distinguish young depressive patients from healthy controls was 0.927. This exploratory study reveals the possible cognitive impairment in young patients with depressive disorders, initially proves the effectiveness of a new method for identifying and evaluating depressive disorders in young people based on cognitive neurocomputing, and provides new ideas for the intelligent identification and evaluation of young people with depressive disorders. In the future, our goal is to further expand the sample size of the study, including people of different age groups, and to expand the generality of the study results. At the same time, we will also optimize the current paradigm and design a new paradigm that can simultaneously reflect the attention, executive function, memory, and information processing speed of patients with depression disorders, and more comprehensively evaluate cognitive impairment in patients with depression disorders. In addition, we will incorporate functional brain neuroimaging techniques to further explore the neurobiological mechanisms that may be involved in this new approach to digital biomarkers.

## Data Availability

The raw data supporting the conclusions of this article will be made available by the authors without undue reservation.
